# Physiological and Biochemical Responses of *Yarrowia lipolytica* to Dehydration Induced by Air-Drying and Freezing

**DOI:** 10.1371/journal.pone.0111138

**Published:** 2014-10-28

**Authors:** Caroline Pénicaud, Sophie Landaud, Frédéric Jamme, Pauline Talbot, Marielle Bouix, Sarrah Ghorbal, Fernanda Fonseca

**Affiliations:** 1 INRA, UMR782 Génie et Microbiologie des Procédés Alimentaires, Thiverval-Grignon, France; 2 AgroParisTech, UMR782 Génie et Microbiologie des Procédés Alimentaires, Thiverval-Grignon, France; 3 Synchrotron SOLEIL, Gif-sur-Yvette, France; Medical College of Georgia, United States of America

## Abstract

Organisms that can withstand anhydrobiosis possess the unique ability to temporarily and reversibly suspend their metabolism for the periods when they live in a dehydrated state. However, the mechanisms underlying the cell’s ability to tolerate dehydration are far from being fully understood. The objective of this study was to highlight, for the first time, the cellular damage to *Yarrowia lipolytica* as a result of dehydration induced by drying/rehydration and freezing/thawing. Cellular response was evaluated through cell cultivability determined by plate counts, esterase activity and membrane integrity assessed by flow cytometry, and the biochemical composition of cells as determined by FT-IR spectroscopy. The effects of the harvesting time (in the log or stationary phase) and of the addition of a protective molecule, trehalose, were investigated. All freshly harvested cells exhibited esterase activity and no alteration of membrane integrity. Cells freshly harvested in the stationary phase presented spectral contributions suggesting lower nucleic acid content and thicker cell walls, as well as longer lipid chains than cells harvested in the log phase. Moreover, it was found that drying/rehydration induced cell plasma membrane permeabilization, loss of esterase activity with concomitant protein denaturation, wall damage and oxidation of nucleic acids. Plasma membrane permeabilization and loss of esterase activity could be reduced by harvesting in the stationary phase and/or with trehalose addition. Protein denaturation and wall damage could be reduced by harvesting in the stationary phase. In addition, it was shown that measurements of loss of membrane integrity and preservation of esterase activity were suitable indicators of loss and preservation of cultivability, respectively. Conversely, no clear effect of freezing/thawing could be observed, probably because of the favorable operating conditions applied. These results give insights into *Y. lipolytica* mechanisms of cellular response to dehydration and provide a basis to better understand its ability to tolerate anhydrobiosis.

## Introduction

In their natural habitats, most living organisms may be periodically subjected to quite intense dehydration, resulting in the state of anhydrobiosis. Organisms that can withstand anhydrobiosis possess the unique ability to temporarily and reversibly suspend their metabolism for periods when environmental conditions are unfavorable [1]. This ability is widely used, mainly in food-related and biotechnology processes that produce or use starters (stabilized microorganisms) that must be efficiently reactivated and functional upon rehydration. However, the mechanisms underlying the cell’s ability to deal with dehydration are far from being fully understood.

From both the genetic and physiological point of view, yeast is a preferred organism for molecular cell biologists because it provides information that is useful in food and applied biotechnology but that is also relevant for other eukaryotes such as mammalian and plant cells [2]. The yeast *Saccharomyces cerevisiae* has been extensively investigated and its response to dehydration has been the subject of many studies [2]–[6]. The dehydration of industrial yeast can be achieved by either drying or freezing. During drying, dehydration occurs due to water removal, whereas during freezing, dehydration occurs due to water solidification. Drying/rehydration and freezing/thawing imply combinations of thermal (heat and cold), osmotic, mechanical and oxidation stress [3], [4], [7]–[9]. The contribution of each stress to the cell’s response is difficult to evaluate, especially since several cell sites can be affected. The plasma membrane is known to be deeply injured: dehydration changes its fluidity [10], [11] and its organization [8], [9], [12], [13], and causes lipid peroxidation [3], [14]–[16]. Due to dehydration, cellular proteins can unfold, aggregate and lose their activity in an irreversible manner [4], [17]. Dehydration can also affect cell wall assembly and further induce wall disruption [7], [18], thus causing cell shape alteration and cell integrity degradation. Dehydration is also thought to cause damage to DNA and nucleic acids, probably by oxidation reactions [3].

Despite our incomplete knowledge about the mechanisms underlying the cell’s response to dehydration, some general statements could be made concerning different microorganisms. First, cultures harvested during the stationary phase generally exhibit better survival than cultures from the log phase [4]. Second, the use of a protective molecule enables the improvement of the survival of microorganisms [4]. Trehalose, a non-reducing disaccharide, is a very well-known protective molecule for yeasts. It has been shown to act as an energy and carbon reserve, to mechanically stabilize proteins and membranes, to prevent oxidative damage by oxygen radicals scavenging, and to protect microorganisms from cold temperatures [19]. Recently, trehalose proved to be an efficient molecule to protect non-conventional yeast *Yarrowia lipolytica* during freeze-drying, a process combining freezing and drying [20]. Formerly known as *Candida*, *Endomycopsis* or *Saccharomycopsis lipolytica*, *Yarrowia lipolytica* is currently taxonomically assigned to the class *Hemiascomycetes* and the family *Dipodascacea* [21]. It is a “generally recognized as safe” (GRAS) microorganism with no adverse effect on humans and has various biotechnological applications [22]–[25] that are of great interest for the bio-industries. It is able to produce organic acids [23], lipases [25] and heterologous proteins [22]. Its major potential is its ability to accumulate large amounts of lipids [26] that can be further extracted to obtain chemical products such as lubricants, adhesives and plastics, as well as biodiesel [24], [27].

*Y. lipolytica* is physiologically very distant from *S. cerevisiae*. It is an obligate aerobe and grows as a mixture of budding cells, pseudohyphae and true hyphae [28]. It has the unique ability among yeasts to metabolize hydrophobic substrates, including n-alkanes, oils, fats and fatty acids [28], [29]. It is frequently isolated from different food media (cheeses, sausages, etc.), as well as from soils, sewage and natural environments such as oil fields [28]. When using sequence analysis of internal transcribed spacers ITS1 and ITS2, forty *Y. lipolytica* strains showed very limited variability among strains [30]. Hence, the study of a single strain can provide interesting insights into the behavior of this species. In addition, its physiology seems closer to that of higher eukaryotes (mammals) than the conventional yeast *S. cerevisiae* in terms of its genome [31] and the proteins involved in vesicular transport [32], thus making it an interesting model to obtain information that can be extended to the human health domain. However, to the best of our knowledge, its response to dehydration induced by air-drying and freezing has not yet been the subject of any investigation, a regrettable lack because a better understanding of this yeast species could be complementary to what is already known about *S. cerevisiae* and could thus increase our understanding of the mechanisms underlying the cell’s ability to tolerate dehydration.

Flow cytometry combined with fluorescent probes is a powerful technique for the rapid analysis of single cells in a mixture by means of light-scattering and fluorescence measurements. It is commonly used to evaluate the viability [33]–[35] and plasma membrane damage [8] of *S. cerevisiae* cells after desiccation stress, to monitor cell damage and to predict fermentation activity of dried yeasts [36], to assess the heterogeneity of heat stress response among individual yeasts [37], as well as to investigate the intracellular physiological events of yeast during yeast storage [38]. Compared to other analytical tools where a single value for each parameter is obtained for the whole population, flow cytometry provides data for every particle detected. Since cells differ in their physiological states, the power of this method lies both in the possibility of determining a wide range of physiological cell parameters at the level of a single cell and in the ability to obtain information about their distribution within cell populations [39].

Cell metabolism and composition can also be explored using FT-IR (Fourier Transformed Infra-Red) spectroscopy, which can provide spectral fingerprints of biological macromolecules such as lipids, proteins, nucleic acids and carbohydrates, and is therefore sensitive to structural and compositional changes in tissues [40], [41]. It has been introduced in microbiology for species identification and strain discrimination [42]–[44] and is now frequently used as a very sensitive and non-destructive method to discriminate between yeasts [45] and to study their biochemical changes [46], [47]. One of the strengths of FT-IR studies is that they can provide data on the metabolic status of whole cells [48]–[50].

Changes in the features of the infrared spectra following the progression of the cell cycle monitored via flow cytometry have been successfully investigated for myeloid leukemia (ML-1) cells [51] and rat fibroblast cells [52]. Hence, flow cytometry and FT-IR are two powerful complementary methods to obtain a general view of metabolism. Their combination could possibly provide a clear picture of the mechanisms involved in yeast stress response.

The objective of this study was to highlight the cellular damage to *Yarrowia lipolytica* under dehydration and following large temperature changes. Cellular response was evaluated through cell cultivability determined by plate counts, esterase activity and membrane integrity assessed by flow cytometry and the biochemical composition of cells as determined by FT-IR spectroscopy. Using these complementary tools, cell plasma membrane permeabilization, protein denaturation, wall damage and oxidation of nucleic acids were shown to be involved in cell sensitivity to drying and rehydration. The benefits of harvesting in the stationary phase and of adding trehalose to improve cell resistance to drying and rehydration could also be confirmed.

## Materials and Methods

### Yeast strain and materials

Yeast strain *Yarrowia lipolytica* CLIB183 was obtained from the International Center for Microbial Resources (CIRM), Grignon, France.

Glucose, glycerol, trehalose and propidium iodide were furnished by Sigma-Aldrich. Sodium chloride, sodium phosphate and aluminum plates were purchased from VWR. Yeast extract, bactopeptones, agar type E, citric acid, Chemchrome V8 (containing carboxyfluorescein di-acetate) and acetone were provided by Organotechnie, Becton Dickinson and Company, Biokar, Acros Organics, AES-Chemunex and Fisher Chemical, respectively.

### Yeast growth conditions

**Figure 1:**
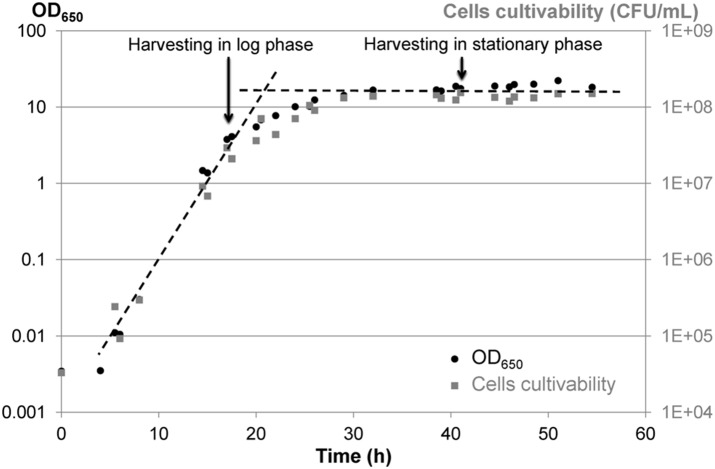
Growth curves of *Yarrowia lipolytica* at 28°C in YPD and indication of harvesting times. Growth curves were followed by DO_650_ (black dots) and cell cultivability (gray boxes, CFU/mL).

Yeast inocula (∼10^7^ cells/mL in YDG (5 g/L Yeast extract, 10 g/L glucose (Dextrose), 20 mL/L Glycerol)) were stored at −80°C and were thawed at ambient temperature for 5 min before inoculation of the pre-culture. Pre-culture was carried out by inoculating 200 µL of cryotube into 20 mL YPD medium (10 g/L Yeast extract, 10 g/L bactoPeptones, 10 g/L glucose (Dextrose)) contained in a 50-mL Erlenmeyer flask. Cells were placed at 28°C in a thermostated orbital shaker (180 rpm) (INFORS Unitron, INFORS SARL, Massy, France) and were grown for 24 h, the time necessary to achieve the stationary phase. The pre-culture was then diluted in fresh YPD medium by a factor of 1/100, and 1 mL of the diluted suspension was inoculated into 100 mL YPD medium contained in a 500-mL Erlenmeyer flask, corresponding to the initial ∼1.5·10^4^ cells/mL. The flask was placed at 28°C on the thermostated orbital shaker (180 rpm) until harvesting. Cells were harvested at the end of the log phase growth (17.5 h culture, i.e., ∼2·10^7^ cells/mL) and in the stationary phase (41.5 h culture, i.e., ∼2·10^8^ cells/mL) (Figure 1). Two complete flasks were pooled for each harvesting in order to obtain enough cells for all subsequent analysis.

### Cell protection and stabilization

For each harvesting, cell pellets were obtained by culture centrifugation (4°C, 15 557 g, 15 min). Cells were then resuspended at 0.5 g/L either in trehalose solution (10% w/v in deionized water) or saline water (8.9 g/L NaCl in deionized water) as neutral diluting medium. This led to a concentration of suspended cells at a rate of ∼2·10^8^ cells/mL for cells harvested in the log phase and ∼1·10^9^ cells/mL for cells harvested in the stationary phase. Each suspension was separated into three fractions as follows: 1.5 mL kept for colony-forming ability count and flow cytometry analysis; 1 mL introduced into a cryotube and frozen in a freezer at −80°C (IK Froilabo Artic 700, Emerainville, France) for 24 h, corresponding to a freezing rate of approximately 4°C/min; 2 mL introduced onto an aluminum plate (diameter: 44 mm) and dried in an incubator (Thermosi SR 3000, Fisher Scientific, Elancourt, France) at 37°C for 22 h. Stabilized cells were not stored. Hence, just after treatment (22 h drying or 24 h freezing), dried or frozen cells were either rehydrated in saline water (9 mL) for 30 min or thawed for 30 min at ambient temperature for analysis.

**Figure 2:**
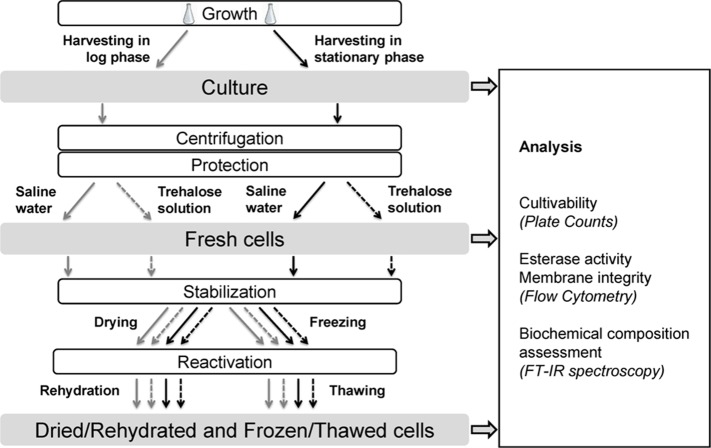
Diagram of the experimental procedure used and the analysis performed on the samples. Cultivability and flow cytometry analysis were performed on ten independent biological replicates. Among these ten biological replicates, three were also devoted to FT-IR analysis.

Figure 2 summarizes the conditions analyzed for each harvesting time (either in exponential growth or stationary phase): culture, cells freshly suspended in saline water or trehalose, dried/rehydrated cells in saline water or trehalose, and frozen/thawed cells in saline water or trehalose. Cell growth, protection and stabilization were independently performed ten times for cultivability evaluation and flow cytometry analysis. Among these ten biological replicates, three were also devoted to FT-IR analysis.

### Cell cultivability

Colony-forming units (CFU/mL) were evaluated by plate counts. After serial decimal dilutions in saline water, cells were plated onto solid YPD agar (10 g/L yeast extract, 10 g/L bactopeptones, 10 g/L glucose, 15 g/L agar type E) and incubated at 28°C for 48 h before cell counting. For each condition, the result corresponded to a geometrical mean of at least three plates and was expressed as log (CFU/mL).

The cultivability loss was calculated as the difference between log (CFU/mL) before and after stabilization by drying or freezing.

### Evaluation of esterase activity and membrane integrity using flow cytometry

Two fluorescent probes, carboxy Fluorescein Di-Acetate (cFDA) and Propidium Iodide (PI), were used as indicators of esterase activity and loss of membrane integrity, respectively [39]. cFDA is an esterase substrate that yields the fluorescent compound carboxy Fluorescein (cF) after internalization with intact membranes and hydrolysis by cellular esterases. cF retained in the cells fluoresces green (at 530 nm) after excitation at 488 nm. The PI probe cannot penetrate within cells with intact membranes but can enter when cell membranes have been damaged, so-called loss of membrane integrity or membrane permeabilization. Inside the cells, it binds to the DNA and fluoresces red (at 615 nm) after excitation at 488 nm.

Before staining, cell suspensions were diluted in McIlvaine buffer (pH 4) to reach approximately 10^6^ cells/mL. The McIlvaine buffer is composed of 0.1 M C_6_H_8_O_7_ (citric acid) and 0.2 M Na_2_HPO_4_ (sodium phosphate). The diluted suspension (940 µL) was supplemented with 10 µL of PI solution (15 mM in distilled water) and 50 µL of Chemchrome V8 (10% v/v in acetone), and then incubated 10 min at 40°C (water bath). ChemChrome V8 was preferred to pure cFDA probe to improve the reliability of the esterase activity measurement [39]. Fluorescence was measured by flow cytometry using a CyFlow Space cytometer (PARTEC SARL, Sainte-Geneviève-des-Bois, France). The cytometer was equipped with a solid blue laser, emitting at 488 nm, and four band-pass filters: a forward-angle light scatter (FSC) combined with a diode collector, a side-angle light scatter (SSC) and two fluorescence signals collected with photomultiplier tubes, a 530-nm band-pass filter (515–545) to collect green fluorescence (FL1 channel), and a 630-nm long-pass filter to collect the red fluorescence (FL2 channel). Analyses were performed using logarithmic gains and specific detector settings, adjusted on a sample of unstained cells, to eliminate cellular autofluorescence. Gating on FSC/SSC was used to discriminate yeasts from the background. Data were collected and analyzed using FloMax software (PARTEC SARL, Sainte-Geneviève-des-Bois, France). This software performed statistical data analysis and indicated numbers and percentages of stained cells determined by each detector: green, red, dual-stained or unstained. An example of raw data (case of dried/rehydrated cells harvested in the stationary phase and suspended in trehalose solution) is shown as Figure S1 to better illustrate all of the features described above.

### Cell biochemical composition assessed by Fourier Transformed Infra-Red (FT-IR) spectroscopy

Infrared absorption measurements were carried out using a Nicolet Magma 750 FT-IR spectrometer (Thermo Fisher Scientific, Madison, WI, USA) equipped with a sample holder allowing measurements in transmission mode and a Nicolet Magma 550 FT-IR spectrometer (Thermo Fisher Scientific, Madison, WI, USA) equipped with a single reflection monolithic diamond Attenuated Total Reflectance (ATR) device (Heated Golden Gate ATR, Specac, Slough, England). Both spectrometers were equipped with a narrow-band mercury/cadmium/telluride (MCT) liquid nitrogen cooled infrared detector and the optical benches were continuously purged with dry air (Balston, Haverhill, MA, USA).

For infrared analyses, cells were washed twice with saline water (10 mL) and then with milliQ water (10 mL; conductivity: 6.2 MΩ cm) and finally centrifuged (4°C, 15 557 g, 5 min) to obtain a pellet that was as dry as possible to avoid contamination effects by water on the spectra. For measurements in transmission mode, the pellet was sandwiched between two CaF_2_ windows (ISP Optics, Riga, Latvia). For measurements in ATR mode, the pellet was deposited on a diamond crystal plate and the movable crystal was further placed in direct contact with the sample. In both cases, it was necessary to wait for five minutes to let the sample dry sufficiently before spectra acquisition.

For each condition, FT-IR spectra of at least three different sandwiches/deposits were recorded in each measurement mode (transmission and ATR), with 128 co-added scans encompassing the mid-IR region from 4000 to 900 cm^−1^. Such a combination of three machine replicas for each of the three biological replicas has been shown to significantly reduce the heterogeneity among results, allowing discrimination of the biological signal from the experimental error [53]. A spectral resolution of 6 cm^−1^ (one point recorded every 3 cm^−1^) was chosen: the infrared band widths analyzed are quite large (width >20 cm^−1^) and, therefore, a spectral resolution of 6 cm^−1^ is adequate to study band intensities and shifts. Spectral display was carried out using OMNIC software (Thermo Fisher Scientific, Madison, WI, USA).

### Statistical analysis

#### Cultivability, esterase activity and membrane integrity data

Two-sample permutation tests were conducted to contrast data concerning cell cultivability, loss of cultivability, green-stained cells, red-stained cells, dual-stained cells and unstained cells. The significance of results was assessed at a 95% confidence level. Two distributions of stained cells in a population were considered to be significantly different when either green- or red-stained cells proved to be significantly different between the two populations. However, when green-stained cells from two populations were significantly different, the red-stained cells were also, and inversely. Hence, when two distributions of stained cells in a population were considered significantly different, it meant that both green- and red-stained cells were significantly different between the two populations.

The correlation between cultivability or loss of cultivability and the stained cells counted by flow cytometry was assessed through linear regression calculation together with the Spearman correlation coefficient and its *p*-value, representing the strength and the significance of the correlation, respectively.

Linear regression was performed using Excel 2007 software (Microsoft Inc., USA). Statistical tests were carried out using R 2.15.1 software [54].

#### FT-IR data

In this study, Principal Component Analysis (PCA) was used as a powerful chemometric method to reveal variances or combinations of variables among multivariate data obtained by FT-IR spectroscopy. To perform PCA, raw spectra were preprocessed using a Savitzky-Golay second derivative (third-degree polynomial and nine smoothing points) [55] and a further unit vector normalization on each region of interest: 2970–2840 cm^−1^, the CH region that provides information about cell lipids; 1715–1575 cm^−1^, the amide I region that provides information about cell proteins; and 1280–940 cm^−1^, the region that provides information about cell carbohydrates and phosphate groups [40], [41]. PCA were carried out on preprocessed spectra to determine whether or not differences had occurred between biological repetitions and to observe which differences had occurred between samples. Spectra obtained in transmission mode were considered separately from those obtained in ATR mode to avoid supplementary processing of ATR spectra, making it possible to compare spectra obtained by the two modes [56]. Transmission infrared revealed a better signal-to-noise ratio in the CH region (2970–2840 cm^−1^) than the ATR approach. In ATR mode, the penetration depth of the evanescent wave (dp), which extends into the sample, is proportional to the wavelength λ [57]:



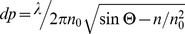
(1)where *n* and *n_0_* are the refraction indices of the sample under study and 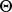 of the crystal, respectively, and is the angle of internal reflection.

This means that the evanescent wave goes deeper (dp) into the sample for the highest wavelength, explaining why ATR reveals a better signal-to-noise ratio in the amide, nucleic acid and carbohydrate regions that correspond to higher wavelengths (8–12 microns) compared to the CH region (2–3 microns). As a consequence, the results presented in the following sections will be those analyzed in transmission mode for the CH region and in ATR mode for the other regions.

Briefly, PCA were performed using nine individual spectra for each sample (three biological replicates combined with three machine replicates), obtained either in transmission mode (results in the CH region) or in ATR mode (results in the other regions). Using such a statistical analysis on individual spectra, differences between samples could be obtained. However, for the sake of clarity and conciseness, all of the score and loading plots of the PCA are not displayed. To illustrate the differences obtained, the averaged adequately preprocessed spectra are preferentially presented. The preprocessings consisted in: (i) baseline offset and linear correction, as well as vector normalization in the CH region; (ii) second-order derivative and vector normalization in the amide I region; (iii) baseline offset and vector normalization in the region of phosphate groups and carbohydrates.

The standard deviations associated with the averaged wavenumbers shown on the figures were also calculated and were not higher than 3 cm^−1^.

Unscrambler X software (version 10.2; CAMO, Norway) was used for spectra preprocessing and PCA.

## Results

### Yeast survival in response to drying and freezing

The cultivability of cells freshly suspended in saline water or trehalose solution and further dried/rehydrated or frozen/thawed was evaluated for cells harvested in both the log and stationary phases. Results are exposed in Table 1 in where superscript letters represent statistical contrasts between samples at the 95% level confidence. Cultivability of freshly suspended cells was 8.3 log (CFU/mL) and 9.1 log (CFU/mL) when harvested in the log and stationary phase, respectively. Cultivability losses were calculated as the differences between cell cultivability expressed in log (CFU/mL) before and after stabilization treatment.

**Table 1:** Cell cultivability and cultivability loss (log (CFU/mL)) after drying/rehydration and freezing/thawing as a function of harvesting time (in the log or stationary phase), and use of protective medium (trehalose solution) or not (saline solution).

Harvestingtime	Suspensionmedium	Treatment	Cultivability (log(CFU/mL))	Cultivability loss (log(CFU/mL))
Log phase	Saline Water	Fresh	8.3±0.3^d^	
		Dried/Rehydrated	7.2±0.4^g^	1.1±0.5^k^
		Frozen/Thawed	8.1±0.3^e^	0.3±0.1^h^
	Trehalosesolution	Fresh	8.3±0.3^d^	
		Dried/Rehydrated	7.7±0.3^f^	0.6±0.2^j^
		Frozen/Thawed	8.1±0.2^e^	0.2±0.1^h^
Stationary phase	Saline Water	Fresh	9.1±0.3^a^	
		Dried/Rehydrated	8.3±0.3^d^	0.8±0.4^j^
		Frozen/Thawed	8.8±0.2^b^	0.3±0.1^h^
	Trehalose solution	Fresh	9.1±0.3^a^	
		Dried/Rehydrated	8.6±0.3^c^	0.4±0.4^i^
		Frozen/Thawed	8.8±0.2^b^	0.2±0.1^h^

Superscript letters represent statistical contrasts between samples at the 95% level confidence.

Cultivability loss of cells dried in saline water was 1.1 log (CFU/mL) and 0.8 log (CFU/mL) when harvested in the log and stationary phase, respectively, and cultivability loss of cells dried in trehalose was 0.6 log (CFU/mL) and 0.4 log (CFU/mL), respectively. Cells harvested in the stationary phase were thus significantly (*p*<0.05) less sensitive to drying/rehydration than cells harvested in the log phase. For both harvesting phases, trehalose significantly reduced (*p*<0.05) cultivability losses by half.

Cells exhibited low sensitivity to freezing/thawing: the cultivability loss of cells was approximately 0.2–0.3 log (CFU/mL). The samples were not significantly discriminated (*p*>0.05), regardless of the harvesting time and the protective molecule. However, as for drying/rehydration, cells harvested in the stationary phase or suspended in trehalose solution tended to be less sensitive to freezing/thawing than cells harvested in the log phase or suspended in saline water, respectively.

### Yeast esterase activity and membrane integrity in response to drying and freezing

**Figure 3:**
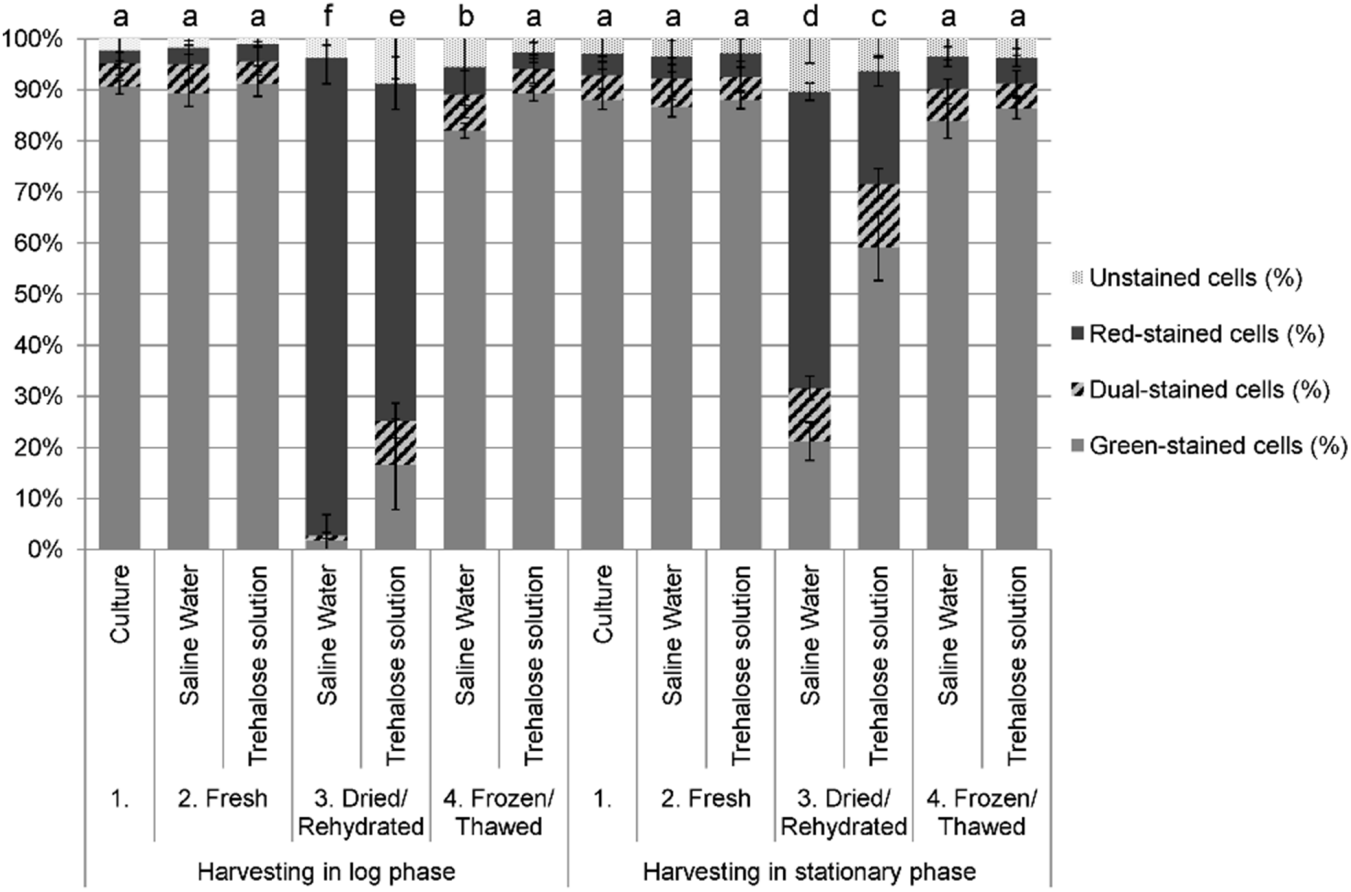
Flow cytometry analysis before and after stabilization treatments. Percentages of green-stained, red-stained, dual-stained and unstained cells of *Yarrowia lipolytica* in the population after drying/rehydration and freezing/thawing as a function of harvesting time (cultured in the log or stationary phase) and use of protective medium (trehalose solution) or not (saline water). Error bars represent experimental standard deviations. Superscript letters represent statistical contrasts between samples at the 95% level confidence.

Two fluorescent probes, carboxy Fluorescein Di-Acetate (cFDA, green fluorescence) and Propidium Iodide (PI, red fluorescence), were used as indicators of esterase activity and loss of membrane integrity, respectively [39]. The fluorescence was measured by flow cytometry after dual staining cFDA/PI of cultured cells, freshly suspended in saline water or trehalose and further dried/rehydrated or frozen/thawed. Green-stained cells reveal esterase activity without loss of membrane integrity; red-stained cells reveal no esterase activity and have lost their membrane integrity; and dual-stained-cells concomitantly reveal esterase activity and loss of their membrane integrity. Figure 3 summarizes the cell responses to drying/rehydration and freezing/thawing in percentages for each category of stained cells, and superscript letters represent statistical contrasts between samples at a 95% level confidence.

Before stabilization, cultured cells and cells freshly suspended in trehalose or saline water were not significantly different (*p*<0.05). Approximately 90% of the cells were green-stained, i.e., they revealed esterase activity without loss of membrane integrity, regardless of the harvesting phase and the suspension medium (no suspension medium (culture), saline water or trehalose solution). Concomitantly, only about 10% of the cells were dual- or red-stained, i.e., presented a loss of esterase activity and/or membrane integrity, meaning that before stabilization, the esterase activity and membrane integrity of the cells were not affected by harvesting and preparation.

After drying/rehydration in saline water, only 21% of cells harvested in the stationary phase were still green-stained, and this was even more drastically decreased to 2% for cells harvested in the log phase. The same samples exhibited 58% and 94% of red-stained cells dried/rehydrated after harvesting in the stationary and log phase, respectively. In all cases, few cells (less than 10%) were dual-stained. The low number of dual-stained cells stated that the dramatic loss of cells with esterase activity occurred concomitantly with a dramatic loss of membrane integrity of these cells. However, harvesting in the stationary phase limited these effects (*p*<0.05). Cells dried in trehalose were also affected but to a lesser extent: when harvested in the stationary phase, 59% of cells were green-stained after drying/rehydration (concomitantly, 22% were red-stained), and this was decreased for cells harvested in the log phase, in which case 17% of the cells were green-stained (and 66% red-stained). The protective effect of trehalose could thus be seen in terms of the preservation of both esterase activity and membrane integrity (*p*<0.05).

On the contrary, freezing did not significantly (*p*>0.05) affect the esterase activity and membrane integrity of cells, with a conservation of more than 90% of green-stained cells after freezing/thawing. Only cells frozen in saline water and harvested in the log phase showed a significant (*p*<0.05), though slight, difference with 82% of green-stained cells and 5% of red-stained cells.

We then investigated whether or not cell cultivability and cultivability losses could be correlated to the numbers of stained cells counted by flow cytometry. Due to the very low number of dual-stained cells obtained in the samples, their inclusion with green- or red-stained cells did not change the obtained correlations, which are presented in Table 2. The green-stained cells (in log (cells/mL)) were compared to the cultivable cells (log (CFU/mL)). A strong positive (ρ = 0.85) and significant (*p* = 2·10^−16^) correlation was obtained. The slope of the linear regression log (green-stained cells) = 0.97·log (CFU/mL) was close to 1, meaning that the number of green-stained cells counted by flow cytometry was very close to that of the colony-forming units. This highlighted the fact that esterase activity preservation measured using cFDA staining was a reliable indicator of cell cultivability.

**Table 2:** Statistical correlations between the stained cells counted by flow cytometry and the cultivable cells determined by plate counts.

	Spearman coefficient		Linear regression
	ρ	*p*-value	
log(green-stained cells) = f (log (CFU/mL))	0.85	2•10^−16^	y = 0.97 x
log(red-stained cells) = f (log (loss of CFU/mL))	0.46	1•10^−5^	y = 0.90 x
log(red-stained+lysed cells) = f (log (loss of CFU/mL))	0.36	1•10^−3^	y = 1.04 x

Green-stained cells are compared to cultivable cells. Red-stained cells and cells missing in the total number of cells before and after treatment (lysed cells) are compared to lost cultivable cells.

The red-stained cells (in log (cells/mL)) were compared to the losses of cultivable cells, calculated as the differences between cell cultivability before and after stabilization treatment (in log (CFU/mL)). A positive (ρ = 0.46) and significant (*p* = 1·10^−5^) correlation was obtained. The linear regression was log (red-stained cells) = 0.90·log (loss of CFU/mL), meaning that the number of red-cells counted by flow cytometry was lower than the loss of the colony-forming units after the drying/rehydration or freezing/thawing process. However, the total cell concentration counted by flow cytometry was lower after the treatment than before, suggesting that some cells suffered lysis and were no longer countable by flow cytometry after treatment. The difference in the total number of cells before and after treatment counted by flow cytometry was calculated to estimate the number of lysed cells during the treatment. This number of lysed cells was added to the number of red-stained cells to be compared to the losses of cultivable cells (Table 2). A positive (ρ = 0.36) and significant (*p* = 1·10^−3^) correlation was also obtained. The slope of the linear regression log(red-stained+lysed cells) = 1.04·log (loss of CFU/mL) was close to 1, confirming that these lysed cells were the cells lacking in the previous correlation. Such results suggest that membrane permeabilization measured using PI staining on cells that did not suffer lysis was a suitable indicator of cell cultivability loss.

### FTIR spectroscopy assessment of biochemical composition in response to drying and freezing

#### In the CH region (2970–2840 cm^−1^)

The spectral range from 2970 to 2840 cm^−1^, attributed to the C–H stretching vibrations, was investigated to identify differences in lipid composition between samples. The lipid contents and the chemical structure of these compounds can be evaluated in this range using peak frequencies at 2956 cm^−1^ (asymmetric stretching vibration of CH_3_ of acyl chains), 2922 cm^−1^ (asymmetric stretching vibration of CH_2_ of acyl chains), 2874 cm^−1^ (symmetric stretching vibration of CH_3_ of acyl chains) and 2852 cm^−1^ (symmetric stretching vibration of CH_2_ of acyl chains) [41]. Lipids can come from cell membranes and from intracellular droplets.

**Figure 4:**
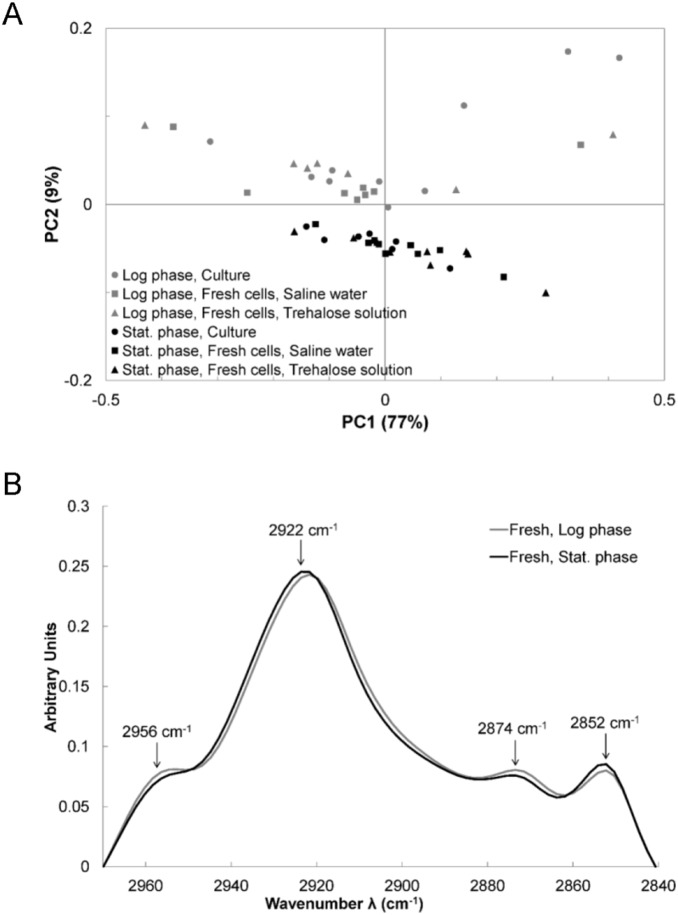
FT-IR analysis in the CH region of fresh cells. Analysis of the 2970–2840 cm^−1^ region of spectra obtained for cells freshly harvested in the log (gray markers and line) or the stationary (black markers and line) phase, directly from culture (dots) and suspended in saline water (boxes) or trehalose (triangles). (A) Score plot of the Principal Component Analysis. (B) Averaged spectra, baseline offset and linearly corrected as well as vector-normalized before being averaged.

The spectra obtained for cells harvested either in the log or stationary phase and suspended either in saline water or in trehalose solution were submitted to PCA. The resulting score plot (Figure 4A) shows the samples (a point represents a sample) plotted in a relevant sub-space determined by principal components PC1 and PC2. Similar samples are grouped together and the score plot is a simple view of the significantly different groups. Although PC1 explained 77% of the variance between data, the samples separated by PC1 did not correspond to different treatments or repetitions. Consequently, PC1 probably represents the biological inter-variability between samples without significance. Following the PC2 axis, the group of cells harvested in the log phase was clearly separated from the group of cells harvested in the stationary phase. However, the points representing the spectra of cultured cells, suspension in saline water or trehalose were not separated (i.e., significantly different) from each other.

Figure 4B presents the averaged spectra of all spectra obtained for cells freshly harvested either in the log or stationary phase. These spectra have been baseline-corrected (offset and linear correction) and vector-normalized before being averaged. The bands ascribed to stretching vibrations of CH_3_ of acyl chains (symmetric: 2874 cm^−1^; asymmetric: 2956 cm^−1^) were more intense for cells harvested in the log phase than for cells harvested in the stationary phase. In contrast, the bands ascribed to the stretching vibrations of CH_2_ of acyl chains (symmetric: 2852 cm^−1^; asymmetric: 2922 cm^−1^) were more intense for cells harvested in the stationary phase than for cells harvested in the log phase. It has been suggested that the intensity of these last two bands was positively related to the amount of lipids in bacterial cells of *Bacillus stearothermophilus* and *Acetobacter aceti* [58].

The same analysis has been done for cells after drying/rehydration and freezing/thawing, in comparison with freshly harvested cells. All features described above were maintained after both stabilization treatments, and no further differences could be observed in comparison with fresh cells (results not shown).

#### In the amide I region (1715–1575 cm^−1^)

**Figure 5:**
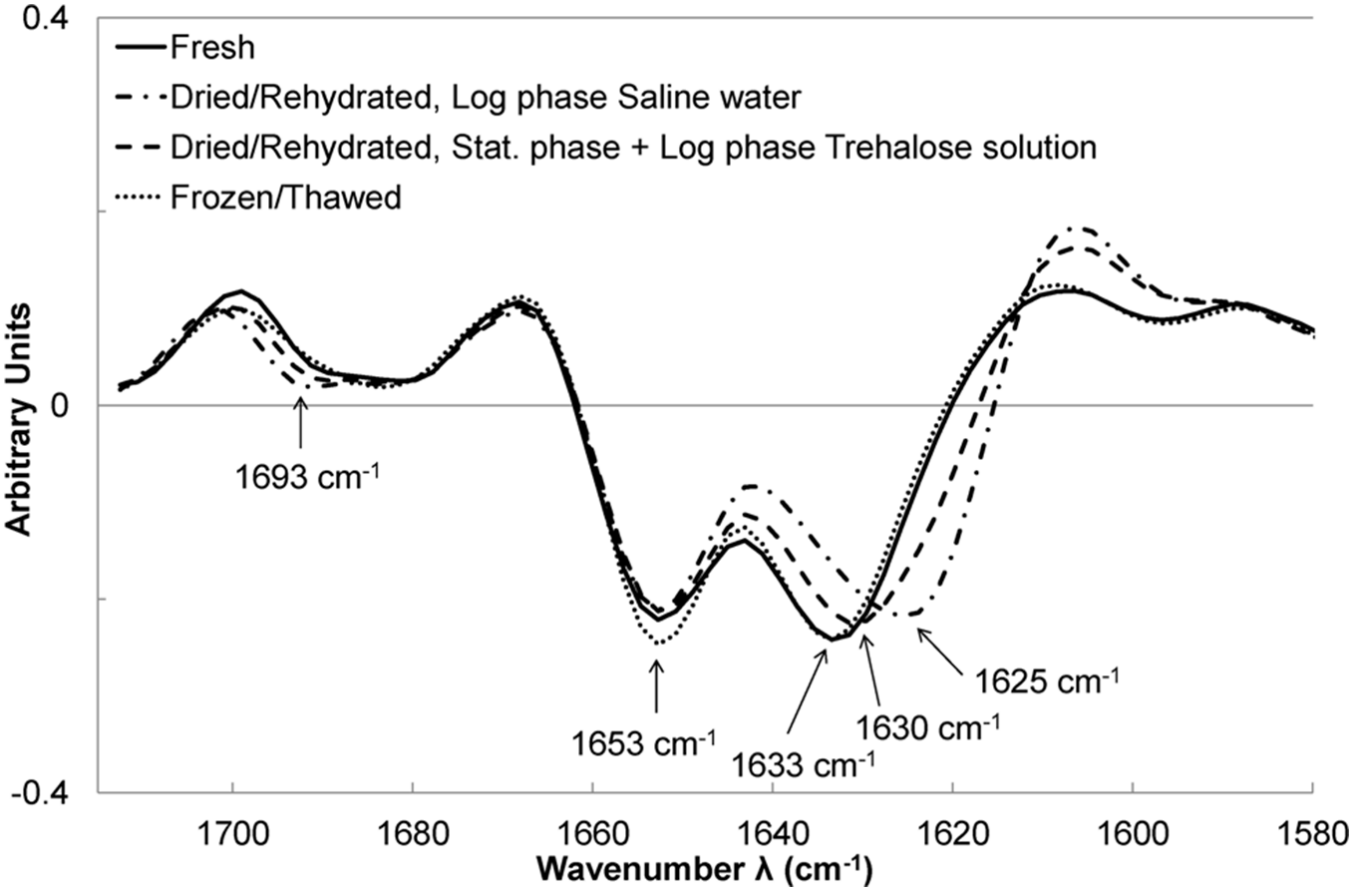
FT-IR analysis in the amide I region before and after stabilization treatments. Averaged second-order derivative and vector-normalized spectra in the 1715–1575 cm^−1^ region of all spectra obtained for freshly harvested cells (solid line) and further dried/rehydrated (dashed line) or frozen/thawed (dotted line). The specific behavior of dried/rehydrated cells after harvesting in the log phase and suspended in saline water (dashed-dotted line) can be observed (dashed line).

The amide I region has been extensively studied in biology due to the information it can provide about protein structure [40]. Figure 5 presents the averages of second-order derivative spectra that have been vector-normalized, i.e., the preprocessed spectra that have been submitted to PCA to reveal differences between samples. Second-order derivatives enhance spectral features such as shoulders and peak shifts. The minima of these derivatives represent the positions of the maxima of corresponding bands on raw spectra. No difference was found between spectra of freshly harvested cells, regardless of the harvesting time (log or stationary phase) or the suspension medium (culture without suspension medium, saline water or trehalose solution), so their spectra were pooled to illustrate the results. Fresh cells presented strong bands at 1653 and 1633 cm^−1^, which are described in the literature as being features of α-helix and β-sheets structures, respectively [40]. Spectra of frozen/thawed cells did not present any significant difference with spectra of fresh cells either (Figure 5). Spectra of dried cells also exhibited these bands. The peak at 1653 cm^−1^ was comparable but the peak at 1633 cm^−1^ was slightly downshifted until 1630 cm^−1^, concomitant with the appearance of a shoulder at 1693 cm^−1^. Such downshift and shoulder appearances have been reported to reveal an increase in the number of β-sheets in the cell proteins and in the number of strands in the β-sheets, linked to protein denaturation [40]. Interestingly, dried/rehydrated cells harvested in the log phase and suspended in saline water were featured by a downshift until 1625 cm^−1^ and a more marked shoulder at 1693 cm^−1^, which means that cells proteins were more affected subsequent to such treatments than subsequent to the other ones (suspension in trehalose, harvesting in stationary phase, freezing).

#### In the region of phosphate groups and carbohydrates (1280–940 cm^−1^)

**Figure 6:**
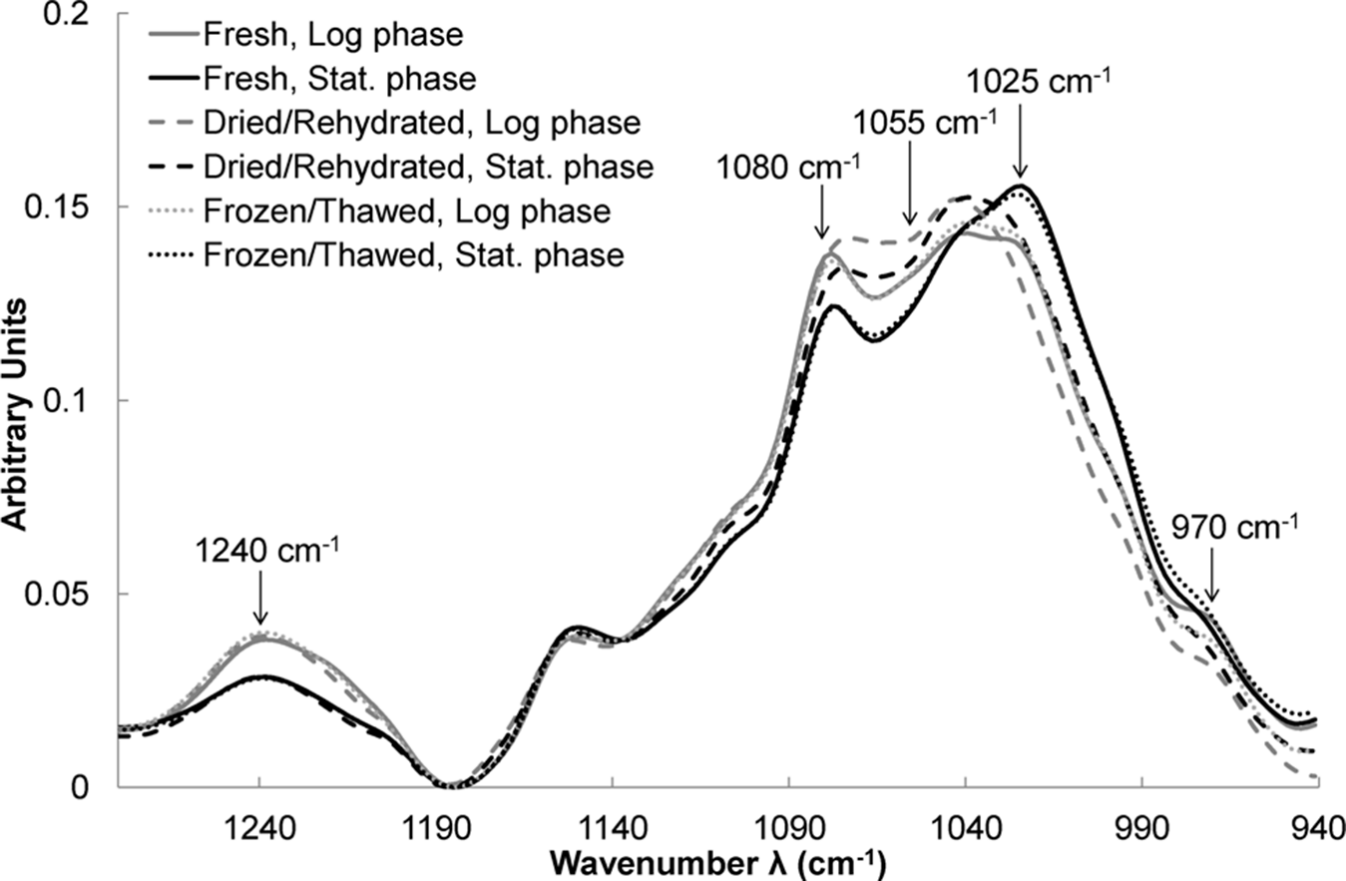
FT-IR analysis in the region of phosphate groups and carbohydrates before and after stabilization treatments. Averaged spectra in the 1280–940 cm^−1^ region of all spectra obtained for freshly harvested cells (solid lines) either in the log (gray lines) or stationary (black lines) phase and further dried/rehydrated (dashed lines) or frozen/thawed (dotted lines). Spectra have been baseline offset and vector-normalized before being averaged.

Strong contrasts were found in the region of phosphate groups and carbohydrates. PCA revealed that cells harvested in the log or stationary phase presented significant differences, as well as dried/rehydrated cells in comparison with fresh cells. Figure 6 shows the averaged spectra baseline offset and vector-normalized for all conditions. Since no differences were evidenced by PCA between cells suspended either in trehalose solution or in saline water, their spectra were pooled for each condition. Cells freshly harvested in the log phase presented more intense bands at 1240 and 1080 cm^−1^, as well as a larger shoulder at 970 cm^−1^ than cells harvested in the stationary phase (Figure 6). Many authors attributed these bands to symmetric (970 and 1080 cm^−1^) and asymmetric (1240 cm^−1^) stretching vibrations of the phosphate groups of nucleic acids [41]. In contrast, cells harvested in the stationary phase presented a more intense band at 1025 cm^−1^ than cells harvested in the log phase (Figure 6). This band has been ascribed to vibrational frequency of -CH_2_OH and to the C-O stretching vibration coupled with C-O bending of the C-OH groups of carbohydrates (glucose, fructose, glycogen) [59]–[62]. Since the yeast cell wall is composed of carbohydrates (mainly glucanes), this band could be attributed to the cell wall contribution to the spectra, as already seen at 1027 cm^−1^ for bacterial isolated walls, as well as intact bacteria in the case of *Bradyrhizobium japonicum* [63].

Drying/rehydration of cell spectral features did not change at 1240 cm^−1^, but the band at 970 cm^−1^ was lower than in fresh cells, thus corroborating nucleic acid injury. Moreover, a strong erosion of the 1055–1080 cm^−1^ region appeared (Figure 6). It has been suggested that such an erosion effect was indicative of oxidative damage to DNA [59], [64]. Drying/rehydration also induced a decrease of the band at 1025 cm^−1^ (Figure 6), suggesting that the cell wall was also affected by the treatment.

In contrast, freezing/thawing did not change spectral features (Figure 6).

## Discussion

This paper aimed at highlighting cellular damage to *Yarrowia lipolytica* as a result of dehydration. The effects of the harvesting time (in the log or stationary phase) and of the addition of a protective molecule, trehalose, were investigated in response to drying/rehydration and freezing/thawing. Cellular response was evaluated through cell cultivability determined by plate counts, esterase activity and membrane integrity assessed by flow cytometry, and the biochemical composition of cells as determined by FT-IR spectroscopy.

### Fresh cells

Freshly harvested cells revealed esterase activity and no membrane permeabilization. No effect of trehalose addition could be observed. FT-IR analysis did not evidence any difference in the cellular protein conformation of fresh cells but, instead, provided complementary information. Cells harvested in the log phase contained more nucleic acids than cells harvested in the stationary phase. It has already been reported that spectral contributions of nucleic acids increased in cells during proliferation [51], [52], [65], [66]. This could be explained by a combination of an increased nucleus-to-cytoplasm ratio and/or by a greater amount of measurable nucleic acids due to less condensed chromatin during replication [65]. Conversely, freshly harvested cells in the stationary phase displayed higher wall spectral contribution than cells harvested in the log phase. This seems consistent with the evidence that cells in the stationary phase have thicker walls than cells in the log phase [67]. In the CH region, which represents lipid composition [41], cells exhibited significant variability. Despite this variability, the CH_2_ spectral contributions were higher for cells harvested in the stationary phase than for cells harvested in the log phase, whereas it was the contrary in the case of CH_3_ spectral contributions. This suggests that cells in the stationary phase contain longer lipid chains than cells in the log phase, which is consistent with the ability of *Y. lipolytica* to produce and store long-chain lipids in the stationary phase [24]. Contradictory results have been obtained in the case of *B. stearothermophilus* and *A. aceti*, i.e., with a maximum lipid content during the exponential growth phase [58]. However, these two bacteria are not able to produce and store long-chain lipids in the stationary phase, perhaps explaining the observed difference with *Y. lipolytica*.

### Frozen/thawed cells

When fresh cells were compared to frozen/thawed cells, no clear and significant differences could be found in their cultivability, membrane integrity, esterase activity or infrared spectral features. However, the differences stated above between fresh cells harvested in the log and stationary phases (lower nucleic acid content and thicker walls, as well as longer lipid chains observed in FT-IR for cells in the stationary phase) were kept after freezing/thawing. Previous studies on *S. cerevisiae* have shown that freezing at a cooling rate lower than 10–20°C/min prevents the formation of intracellular ice and thus preserves cell viability after thawing [68], [69]. In this study, *Y. lipolytica* cells were frozen at approximately 4°C/min. It can thus be hypothesized that cells were frozen/thawed in favorable operating conditions, explaining their similarity to fresh cells.

### Dried/Rehydrated cells

The differences observed between fresh cells harvested in the log and stationary phases were still observed after drying/rehydration. Moreover, this quite drastic treatment induced other contrasted differences in plasma membrane, cellular proteins, nucleic acids and cell wall.

#### Membrane permeabilization

Membrane permeabilization measured by PI staining proved to be correlated to cultivability loss, highlighting the crucial role of membranes in the resistance to dehydration/rehydration. The permeabilization was lower for cells harvested in the stationary phase than in the log phase, and also for cells suspended in trehalose solution than in saline water, regardless of the harvesting time. In accordance with previously reported studies, cells in the stationary phase have more resistant membranes than actively dividing cells that contain budding yeasts [4]. On the other hand, the protective effect of trehalose on the membrane has frequently been observed [19]; trehalose is able to interact with phospholipid polar head groups, providing steric reinforcement, especially following water removal. However, FT-IR measurements in the CH region that represents lipid composition [41] did not make it possible to observe any effect in this study. This can be ascribed to the contribution of other membranes (nucleus, mitochondria, etc.) present in *Y. lipolytica* cells, which may be less affected by dehydration. Furthermore, it is an oleaginous yeast, i.e., capable of accumulating lipid droplets that are not necessarily affected by dehydration. Contrary to PI staining, which is membrane-specific, the FT-IR spectroscopy measurement is a global measurement. Consequently, all cell lipids are estimated together. If only the plasma membrane is affected, this can explain why it was not revealed by FT-IR measurement (fall short).

#### Protein damage

Loss of esterase activity was observed concomitant with protein denaturation, suggesting that protein denaturation was linked to esterase activity loss. Proteins from cells harvested in the log phase and suspended in trehalose solution were less affected than those from cells in saline water. This suggests a protective effect of trehalose on proteins during drying/rehydration. This is consistent with a well-known action mode of trehalose, enabling it to preserve protein structures by replacing water removed during dehydration [19]. Cells harvested in the stationary phase were less affected than in the log phase. The protective effect of trehalose on esterase activity preservation was also significant, but concomitant preservation of protein conformation could not be observed in the stationary phase. This result can be explained by either the preservation of protein conformation or an insufficient FT-IR sensitivity for detecting a low degree of protein denaturation. Such result highlights the complementarities of flow cytometry and FT-IR spectroscopy for investigating the state of cell proteins.

#### Nucleic acids injury

A strong erosion effect appeared on nucleic acid bands at 1055–1080 cm^−1^ after drying/rehydration. Similar erosion has been observed in spectra of oxidatively damaged DNA from breast cancer cells [64]. Such effects on malignant non-Hodgkin’s lymphoma cells spectra were also attributed to oxidative DNA damage [59]. It has already been reported that drying and rehydration induced oxidative stress and damage to nucleic acids in plants [3]. Hence, our findings support the correlation between erosion of the 1055–1080 cm^−1^ region and oxidative damage to nucleic acids. Neither a change in the harvesting time nor the addition of trehalose could prevent this injury. In the literature, the protective effect of trehalose against oxidative damage has been suggested for *S. cerevisiae* [19], but was not confirmed in this study. *Y. lipolytica* may be more sensitive to oxidation than *S. cerevisiae* due to its strictly aerobic metabolism that thus produces many reactive oxygen species in its respiratory chains. Interestingly, the behavior of *Y. lipolytica* seemed closer to the behavior of human cells (breast cancer and non-Hodgkin’s lymphoma) than to that of *S. cerevisiae*. It has already been suggested that the physiology of *Y. lipolytica* was closer to that of higher eukaryotes (mammals) than the conventional yeast *S. cerevisiae* with regard to its genome [31] and the proteins involved in vesicular transport [32]. However, a full study devoted to proving such a similarity with regard to oxidative response to drying/rehydration would be necessary. Such a study could consider the possibility of *Y. lipolytica* as a good model for mammal stress response, especially because oxidative stress on DNA has been shown to be strongly involved in human cancer [70] and neurodegenerative diseases [71].

#### Wall damage

The cell wall was affected, independently of the harvesting time. This can easily be explained by mechanical constraints that the cell has to cope with during dehydration and rehydration [7], [18]. Trehalose also failed to prevent this damage.

## Conclusions

This study provided insights into *Y. lipolytica*’s cellular response to dehydration and established the basis to better understand its ability to tolerate anhydrobiosis. It would be particularly useful to further investigate protein alteration as a key mechanism for explaining cell damage following dehydration/rehydration. Flow cytometry and FT-IR spectroscopy provided complementary information about membrane permeabilization, protein denaturation, and the degradation of cell wall and nucleic acids, thus allowing better explanations of the mechanisms involved in cell injury during drying and rehydration. Esterase activity was clearly related to the preservation of protein conformation and appeared as a suitable indicator of cell survival. Whereas trehalose made it possible to reduce cultivability losses at the log phase by preserving protein conformation, its protective mechanism in the stationary phase still needs to be elucidated. More in-depth investigation of the cellular response to oxidative stress in comparison to mammalian cells also appears to be of major interest.

## Supporting Information

Figure S1 **Example of flow cytometry raw data.** Case of dried/rehydrated cells harvested in the stationary phase and suspended in trehalose solution. (A) Parameter check on unstained cells: FSC and SSC measurements define gating region R1, and cells autofluorescence is eliminated from regions RN1 (green fluorescence) and RN2 (red fluorescence) by adjusting gain parameters (as seen by the surrounding solid gray line). (B) Analysis of stained cells: all parameters defined in (A) are kept and compensation parameters (as seen by the surrounding dotted gray line) are introduced to correct the influence of red fluorescence on green fluorescence. All parameters seen by gray surroundings on (A) and (B) remain constant, regardless of the sample under analysis. For each analysis (B), the percentages of green-stained, dual-stained, red-stained and unstained cells are observed on the FL2 = f (FL1) graph and the values are extracted from the table below the graph (as seen by the surrounding solid black line). These values are the ones used for statistical analysis and shown on Figure 3.
(TIFF)

## References

[1] Crowe JH, Hoekstra FA, Crowe LM (1992) Anhydrobiosis. Annu Rev Physiol 54: 579–599. 1562184 10.1146/annurev.ph.54.030192.003051

[2] Hohmann S, Mager WH (2010) Yeast Stress Responses. New-York: Springer. 389 p.

[3] França MB, Panek AD, Eleutherio ECA (2007) Oxidative stress and its effects during dehydration. Comp Biochem Physiol A Mol Integr Physiol 146: 621–631. 16580854 10.1016/j.cbpa.2006.02.030

[4] Fu N, Chen XD (2011) Towards a maximal cell survival in convective thermal drying processes. Food Res Int 44: 1127–1149.

[5] Beker M, Rapoport A (1987) Conservation of yeasts by dehydration. Adv Biochem Eng/Biotechnol 35: 127–171.

[6] Poirier I, Maréchal PA, Richard S, Gervais P (1999) *Saccharomyces cerevisiae* viability is strongly dependant on rehydration kinetics and the temperature of dried cells. J Appl Microbiol 86: 87–92. 10030013 10.1046/j.1365-2672.1999.00638.x

[7] Ando A, Nakamura T, Murata Y, Takagi H, Shima J (2007) Identification and classification of genes required for tolerance to freeze-thaw stress revealed by genome-wide screening of *Saccharomyces cerevisiae* deletion strains. FEMS Yeast Res 7: 244–253. 16989656 10.1111/j.1567-1364.2006.00162.x

[8] Simonin H, Beney L, Gervais P (2007) Sequence of occurring damages in yeast plasma membrane during dehydration and rehydration: Mechanisms of cell death. Biochim Biophys Acta-Biomembranes 1768: 1600–1610. 10.1016/j.bbamem.2007.03.017 17466936

[9] Lemetais G, Dupont S, Beney L, Gervais P (2012) Air-drying kinetics affect yeast membrane organization and survival. Appl Microbiol Biotechnol 96: 471–480. 22552899 10.1007/s00253-012-4014-3

[10] Beney L, Gervais P (2001) Influence of the fluidity of the membrane on the response of microorganisms to environmental stresses. Appl Microbiol Biotechnol 57: 34–42. 11693931 10.1007/s002530100754

[11] Guyot S, Ferret E, Gervais P (2006) Yeast survival during thermal and osmotic shocks is related to membrane phase change. J Agric Food Chem 54: 8450–8455. 17061820 10.1021/jf0620158

[12] Dupont S, Beney L, Ritt J-F, Lherminier J, Gervais P (2010) Lateral reorganization of plasma membrane is involved in the yeast resistance to severe dehydration. Biochim Biophys Acta-Biomembranes 1798: 975–985. 10.1016/j.bbamem.2010.01.015 20116363

[13] Zheng D, Zhang K, Gao K, Liu Z, Zhang X, et al (2013) Construction of novel *Saccharomyces cerevisiae* strains for bioethanol active dry yeast (ADY) production. PLoS One 8: e85022. 24376860 10.1371/journal.pone.0085022 PMC3871550

[14] Espindola AD, Gomes DS, Panek AD, Eleutherio ECA (2003) The role of glutathione in yeast dehydration tolerance. Cryobiology 47: 236–241. 14697735 10.1016/j.cryobiol.2003.10.003

[15] Pereira ED, Panek AD, Eleutherio ECA (2003) Protection against oxidation during dehydration of yeast. Cell Stress Chaperones 8: 120–124. 14627197 10.1379/1466-1268(2003)008<0120:paoddo>2.0.co;2 PMC514863

[16] Garre E, Raginel F, Palacios A, Julien A, Matallana E (2010) Oxidative stress responses and lipid peroxidation damage are induced during dehydration in the production of dry active wine yeasts. Int J Food Microbiol 136: 295–303. 19914726 10.1016/j.ijfoodmicro.2009.10.018

[17] Prestrelski SJ, Tedeschi N, Arakawa T, Carpenter JF (1993) Dehydration-Induced Conformational Transitions in Proteins and Their Inhibition by Stabilizers. Biophys J 65: 661–671. 7693001 10.1016/S0006-3495(93)81120-2 PMC1225768

[18] Levin DE (2011) Regulation of Cell Wall Biogenesis in *Saccharomyces cerevisiae*: The Cell Wall Integrity Signaling Pathway. Genetics 189: 1145–1175. 22174182 10.1534/genetics.111.128264 PMC3241422

[19] Elbein AD, Pan YT, Pastuszak I, Carroll D (2003) New insights on trehalose: a multifunctional molecule. Glycobiology 13: 17R–27R. 10.1093/glycob/cwg047 12626396

[20] Połomska X, Wojtatowicz M, Zarowska B, Szołtysik M, Chrzanowska J (2012) Freeze-Drying Preservation of Yeast Adjunct Cultures for Cheese Production. Polish J Food Nutr Sci 62: 143–150.

[21] Kurtzman CP (2011) Yarrowia. In: Kurtzman CP, Fell JW, Boekhout T, editors. The Yeasts: A Taxonomic Study, Volume 2. Amsterdam, the Netherlands: Elsevier. 927–930.

[22] Madzak C, Gaillardin C, Beckerich J-M (2004) Heterologous protein expression and secretion in the non-conventional yeast *Yarrowia lipolytica*: a review. J Biotechnol 109: 63–81. 15063615 10.1016/j.jbiotec.2003.10.027

[23] Finogenova TV, Morgunov IG, Kamzolova SV, Chernyavskaya OG (2005) Organic acid production by the yeast *Yarrowia lipolytica*: A review of prospects. Appl Biochem Microbiol 41: 418–425. 16240644

[24] Papanikolaou S, Aggelis G (2010) *Yarrowia lipolytica*: A model microorganism used for the production of tailor-made lipids. Eur J Lipid Sci Technol 112: 639–654.

[25] Fickers P, Marty A, Nicaud JM (2011) The lipases from *Yarrowia lipolytica*: Genetics, production, regulation, biochemical characterization and biotechnological applications. Biotechnol Adv 29: 632–644. 21550394 10.1016/j.biotechadv.2011.04.005

[26] Beopoulos A, Nicaud JM, Gaillardin C (2011) An overview of lipid metabolism in yeasts and its impact on biotechnological processes. Appl Microbiol Biotechnol 90: 1193–1206. 21452033 10.1007/s00253-011-3212-8

[27] Beopoulos A, Cescut J, Haddouche R, Uribelarrea J-L, Molina-Jouve C, et al (2009) *Yarrowia lipolytica* as a model for bio-oil production. Prog Lipid Res 48: 375–387. 19720081 10.1016/j.plipres.2009.08.005

[28] Barth G, Gaillardin C (1997) Physiology and genetics of the dimorphic fungus *Yarrowia lipolytica* . Fems Microbiol Rev 19: 219–237. 9167256 10.1111/j.1574-6976.1997.tb00299.x

[29] Fickers P, Benetti P-H, Waché Y, Marty A, Mauersberger S, et al (2005) Hydrophobic substrate utilisation by the yeast *Yarrowia lipolytica*, and its potential applications. FEMS Yeast Res 5: 527–543. 15780653 10.1016/j.femsyr.2004.09.004

[30] Naumova ES, Serpova EV, Naumov GI (2010) Genome variability of the yeast *Yarrowia lipolytica* . Microbiology 79: 229–236.

[31] Casaregola S, Neuveglise C, Lepingle A, Bon E, Feynerol C, et al (2000) Genomic Exploration of the Hemiascomycetous Yeasts: 17. *Yarrowia lipolytica* . FEBS Lett 487: 95–100. 11152892 10.1016/s0014-5793(00)02288-2

[32] Swennen D, Beckerich J-M (2007) *Yarrowia lipolytica* vesicle-mediated protein transport pathways. BMC Evol Biol 7: 219. 17997821 10.1186/1471-2148-7-219 PMC2241642

[33] Rodríguez-Porrata B, Novo M, Guillamón J, Rozès N, Mas a, et al (2008) Vitality enhancement of the rehydrated active dry wine yeast. Int J Food Microbiol 126: 116–122. 18619697 10.1016/j.ijfoodmicro.2008.05.016

[34] Singh J, Kumar D, Ramakrishnan N, Singhal V, Jervis J, et al (2005) Transcriptional response of *Saccharomyces cerevisiae* to desiccation and rehydration. Appl Environ Microbiol 71: 8752–8763. 16332871 10.1128/AEM.71.12.8752-8763.2005 PMC1317403

[35] López-martínez G, Pietrafesa R, Romano P, Cordero-otero R, Capece A (2013) Genetic improvement of *Saccharomyces cerevisiae* wine strains for enhancing cell viability after desiccation stress. Yeast 30: 319–330. 23576041 10.1002/yea.2952

[36] Attfield PV, Kletsas S, Veal DA, van Rooijen R, Bell PJL (2000) Use of flow cytometry to monitor cell damage and predict fermentation activity of dried yeasts. J Appl Microbiol 89: 207–214. 10971752 10.1046/j.1365-2672.2000.01100.x

[37] Attfield P V, Choi HY, Veal DA, Bell PJL (2001) Heterogeneity of stress gene expression and stress resistance among individual cells of *Saccharomyces cerevisiae* . Mol Microbiol 40: 1000–1008. 11401706 10.1046/j.1365-2958.2001.02444.x

[38] Gabier AC, Gourdon P, Reitz J, Leveau JY, Bouix M (2005) Intracellular physiological events of yeast *Rhodotorula glutinis* during storage at +4°C. Int J Food Microbiol 105: 97–109. 16253365 10.1016/j.ijfoodmicro.2005.02.005

[39] Díaz M, Herrero M, García LA, Quirós C (2010) Application of flow cytometry to industrial microbial bioprocesses. Biochem Eng J 48: 385–407.

[40] Barth A (2007) Infrared spectroscopy of proteins. Biochim Biophys Acta-Bioenergetics 1767: 1073–1101. 10.1016/j.bbabio.2007.06.004 17692815

[41] Movasaghi Z, Rehman S, ur Rehman DI (2008) Fourier Transform Infrared (FTIR) Spectroscopy of Biological Tissues. Appl Spectrosc Rev 43: 134–179.

[42] Helm D, Labischinski H, Schallehn G, Naumann D (1991) Classification and identification of bacteria by Fourier-Transform Infrared-Spectroscopy. J Gen Microbiol 137: 69–79. 1710644 10.1099/00221287-137-1-69

[43] Naumann D, Fijala V, Labischinski H, Giesbrecht P (1988) The rapid differentiation and identification of pathogenic bacteria using Fourier transform infrared spectroscopic and multivariate statistical analysis. J Mol Struct 174: 165–170.

[44] Naumann D, Helm D, Labischinski H (1991) Microbiological characterizations by FT-IR spectroscopy. Nature 351: 81–82. 1902911 10.1038/351081a0

[45] Adt I, Kohler A, Gognies S, Budin J, Sandt C, et al (2010) FTIR spectroscopic discrimination of *Saccharomyces cerevisiae* and *Saccharomyces bayanus* strains. Can J Microbiol 56: 793–801. 20921989 10.1139/w10-062

[46] Galichet a, Sockalingum GD, Belarbi A, Manfait M (2001) FTIR spectroscopic analysis of *Saccharomyces cerevisiae* cell walls: study of an anomalous strain exhibiting a pink-colored cell phenotype. FEMS Microbiol Lett 197: 179–186. 11313132 10.1111/j.1574-6968.2001.tb10601.x

[47] Burattini E, Cavagna M, Dell’Anna R, Malvezzi Campeggi F, Monti F, et al (2008) A FTIR microspectroscopy study of autolysis in cells of the wine yeast *Saccharomyces cerevisiae* . Vib Spectrosc 47: 139–147.

[48] Corte L, Rellini P, Roscini L, Fatichenti F, Cardinali G (2010) Development of a novel, FTIR (Fourier transform infrared spectroscopy) based, yeast bioassay for toxicity testing and stress response study. Anal Chim Acta 659: 258–265. 20103133 10.1016/j.aca.2009.11.035

[49] Jamme F, Vindigni JD, Mechin V, Cherifi T, Chardot T, et al (2013) Single Cell Synchrotron FT-IR Microspectroscopy Reveals a Link between Neutral Lipid and Storage Carbohydrate Fluxes in *S. cerevisiae* . PLoS One 8: e74421. 24040242 10.1371/journal.pone.0074421 PMC3770668

[50] Correa-García S, Bermúdez-Moretti M, Travo A, Déléris G, Forfar I (2014) FTIR spectroscopic metabolome analysis of lyophilized and fresh *Saccharomyces cerevisiae* yeast cells. Anal Methods 6: 1855.

[51] Boydston-White S, Gopen T, Houser S, Bargonetti J, Diem M (1999) Infrared spectroscopy of human tissue. V. Infrared spectroscopic studies of myeloid leukemia (ML-1) cells at different phases of the cell cycle. Biospectroscopy 5: 219–227. 10478952 10.1002/(SICI)1520-6343(1999)5:4<219::AID-BSPY2>3.0.CO;2-O

[52] Pacifico A, Chiriboga LA, Lasch P, Diem M (2003) Infrared spectroscopy of cultured cells - II. Spectra of exponentially growing, serum-deprived and confluent cells. Vib Spectrosc 32: 107–115.

[53] Roscini L, Corte L, Antonielli L, Rellini P, Fatichenti F, et al (2010) Influence of cell geometry and number of replicas in the reproducibility of whole cell FTIR analysis. Analyst 135: 2099–2105. 20523945 10.1039/c0an00127a

[54] R Core Team (2012) R: A language and environment for statistical computing. R Foundation for Statistical Computing, Vienna, Austria. ISBN 3-900051-07-0, URL http://www.r-project.org/.

[55] Savitzky A, Golay MJE (1964) Smoothing and Differentiation of Data by Simplified Least Squares Procedures. Anal Chem 36: 1627–1639.

[56] Chalmers JM, Griffiths PR (2002) Handbook of vibrational spectroscopy, Volume 2. Chichester: John Wiley & Sons, Ltd. 1091 p.

[57] Harrick NJ (1967) Internal Reflection Spectroscopy. New York: Wiley Interscience. 327 p.

[58] Ede SM, Hafner LM, Fredericks PM (2004) Structural changes in the cells of some bacteria during population growth: An FT-IR/ATR study. Appl Spectrosc 53: 317–322. 10.1366/000370204322886672 15035713

[59] Andrus PG, Strickland RD (1998) Cancer grading by Fourier transform infrared spectroscopy. Biospectroscopy 4: 37–46. 9547013 10.1002/(sici)1520-6343(1998)4:1<37::aid-bspy4>3.0.co;2-p

[60] Mordechai S, Mordehai J, Ramesh J, Levi C, Huleihel M, et al (2001) Application of FTIR microspectroscopy for the follow-up of childhood leukemia chemotherapy. Subsurface and Surface Sensing Technologies and Applications Iii. 4491: 243–250.

[61] Huleihel M, Salman A, Erukhimovitch V, Ramesh J, Hammody Z, et al (2002) Novel spectral method for the study of viral carcinogenesis in vitro. J Biochem Biophys Methods 50: 111–121. 11741700 10.1016/s0165-022x(01)00177-4

[62] Corte L, Antonielli L, Roscini L, Fatichenti F, Cardinali G (2011) Influence of cell parameters in Fourier transform infrared spectroscopy analysis of whole yeast cells. Analyst 136: 2339–2349. 21494743 10.1039/c0an00515k

[63] Zeroual W, Choisy C, Doglia SM, Bobichon H, Angiboust J, et al (1994) Monitoring of bacterial growth and structural analysis as probed by FT-IR spectroscopy. Biochim Biophys acta 1222: 171–178. 8031853 10.1016/0167-4889(94)90166-x

[64] Malins DC, Polissar NL, Nishikida K, Holmes EH, Gardner HS, et al (1995) The Etiology and Prediction of Breast-Cancer - Fourier Transform-Infrared Spectroscopy Reveals Progressive Alterations in Breast DNA Leading to a Cancer-Like Phenotype in a High Proportion of Normal Women. Cancer 75: 503–517. 7812921 10.1002/1097-0142(19950115)75:2<503::aid-cncr2820750213>3.0.co;2-0

[65] Chiriboga L, Yee M, Diem M (2000) Infrared spectroscopy of human cells and tissue. Part VI: A comparative study of histopathology and infrared microspectroscopy of normal, cirrhotic, and cancerous liver tissue. Appl Spectrosc 54: 1–8.

[66] Krafft C, Sobottka SB, Gabriele SB, Salzer R (2004) Analysis of human brain tissue, brain tumors and tumor cells by infrared spectroscopic mapping. Analyst 129: 921–925. 15457324 10.1039/b408934k

[67] Smith AE, Zhang ZB, Thomas CR, Moxham KE, Middelberg APJ (2000) The mechanical properties of *Saccharomyces cerevisiae* . Proc Natl Acad Sci USA 97: 9871–9874. 10963659 10.1073/pnas.97.18.9871 PMC27610

[68] Mazur P, Schmidt JJ (1968) Interactions of cooling velocity, temperature, and warming velocity on the survival of frozen and thawed yeast. Cryobiology 5: 1–17. 5760041 10.1016/s0011-2240(68)80138-5

[69] Seki S, Kleinhans FW, Mazur P (2009) Intracellular ice formation in yeast cells vs. cooling rate: predictions from modeling vs. experimental observations by differential scanning calorimetry. Cryobiology 58: 157–165. 19118541 10.1016/j.cryobiol.2008.11.011 PMC3760380

[70] Kardeh S, Ashkani-Esfahani S, Alizadeh AM (2014) Paradoxical action of reactive oxygen species in creation and therapy of cancer. Eur J Pharmacol 735: 150–168. 24780648 10.1016/j.ejphar.2014.04.023

[71] Gan L, Johnson JA (2014) Oxidative damage and the Nrf2-ARE pathway in neurodegenerative diseases. Biochim Biophys Acta 1842: 1208–1218. 24382478 10.1016/j.bbadis.2013.12.011

