# Supplementary figures and images for: Physiological and Biochemical Responses of Yarrowia lipolytica to Dehydration Induced by Air-Drying and Freezing
Source: PLoS One. 2014 Oct 28;9(10):e111138. doi: 10.1371/journal.pone.0111138 (PMC4211883; doi:10.1371/journal.pone.0111138)

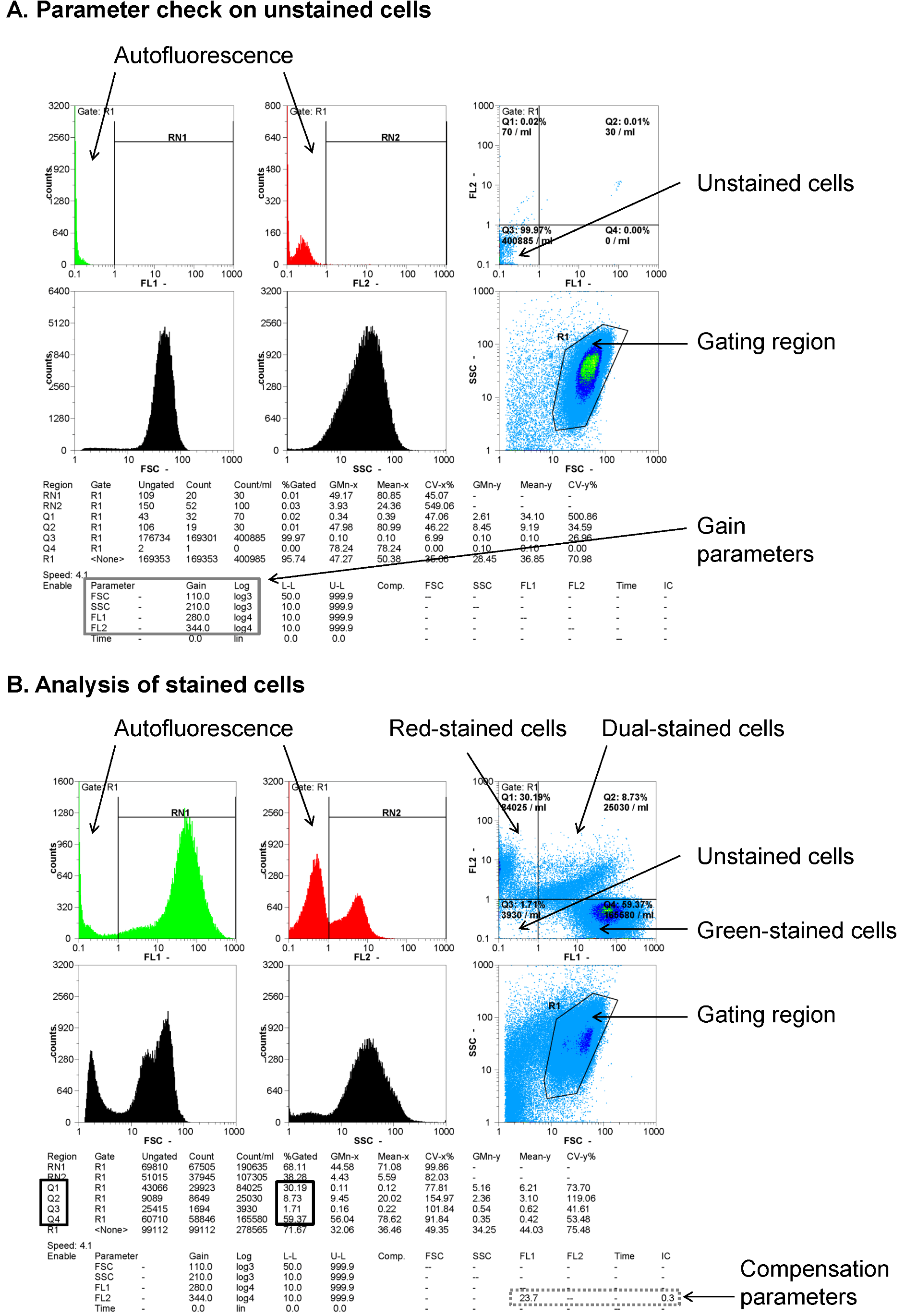

Supplement: Figure S1 — Example of flow cytometry raw data. Case of dried/rehydrated cells harvested in the stationary phase and suspended in trehalose solution. (A) Parameter check on unstained cells: FSC and SSC measurements define gating region R1, and cells autofluorescence is eliminated from regions RN1 (green fluorescence) and RN2 (red fluorescence) by adjusting gain parameters (as seen by the surrounding solid gray line). (B) Analysis of stained cells: all parameters defined in (A) are kept and compensation parameters (as seen by the surrounding dotted gray line) are introduced to correct the influence of red fluorescence on green fluorescence. All parameters seen by gray surroundings on (A) and (B) remain constant, regardless of the sample under analysis. For each analysis (B), the percentages of green-stained, dual-stained, red-stained and unstained cells are observed on the FL2 = f (FL1) graph and the values are extracted from the table below the graph (as seen by the surrounding solid black line). These values are the ones used for statistical analysis and shown on Figure 3. (TIFF) [file pone.0111138.s001.tiff]
